# Hepatitis B Virus-Related Cirrhosis and Hepatocellular Carcinoma Hospital Discharge Rates from 2005 to 2021 in Spain: Impact of Universal Vaccination

**DOI:** 10.3390/vaccines12111254

**Published:** 2024-11-04

**Authors:** Angela Domínguez, Ana Avellón, Victoria Hernando, Núria Soldevila, Eva Borràs, Ana Martínez, Conchita Izquierdo, Núria Torner, Carles Pericas, Cristina Rius, Pere Godoy

**Affiliations:** 1 Department of Medicine, Universidad de Barcelona, 08036 Barcelona, Spain; angela.dominguez@ub.edu (A.D.); eva.borras@gencat.cat (E.B.); nuriatorner@ateneu.ub.edu (N.T.); cpericas@aspb.cat (C.P.); 2 CIBER Epidemiología y Salud Pública (CIBERESP), Instituto de Salud Carlos III, 28029 Madrid, Spain; aavellon@isciii.es (A.A.); a.martinez@gencat.cat (A.M.); crius@aspb.cat (C.R.); pere.godoy@gencat.cat (P.G.); 3 Hepatitis Unit, National Centre of Microbiology, Instituto de Salud Carlos III, 28222 Madrid, Spain; 4 Centro Nacional de Epidemiología, Instituto de Salud Carlos III, 28029 Madrid, Spain; vhernando@isciii.es; 5 CIBER Enfermedades Infecciosas (CIBERINFEC), Instituto de Salud Carlos III, 28029 Madrid, Spain; 6 Agència de Salut Pública de Catalunya, 08005 Barcelona, Spain; conchita.izquierdo@gencat.cat; 7 Agència de Salut Pública de Barcelona, 08023 Barcelona, Spain; 8 Institut de Recerca de l’Hospital de la Santa Creu i Sant Pau (IRB Sant Pau), 08041 Barcelona, Spain; 9 Department MELIS-UPF, Universitat Pompeu Fabra, 08002 Barcelona, Spain; 10 Institut de Recerca Biomédica de Lleida (IRBLleida), 25006 Lleida, Spain

**Keywords:** hepatitis B, hepatocellular carcinoma, cirrhosis, impact evaluation, vaccination

## Abstract

**Background:** The main consequences of chronic hepatitis B virus (HBV) infections are cirrhosis and hepatocellular carcinoma (HCC), both associated with frequent hospitalization. The aim of this study was to analyze the impact of universal HBV vaccination in Spain on chronic HBV-related hospital discharges from 2005 to 2021. **Methods:** Using data from the Minimum Basic Data Set of the Spanish National Health System, we calculated the hospital discharge rate ratio (HDRR) and 95% confidence interval (CI) values for chronic HBV-related discharges between 2005 and 2021. For comparative purposes, we calculated the HDRR and 95% confidence interval (CI) values for the early (2005–2013) and later (2014–2021) periods and the vaccinated compared with unvaccinated cohorts for the 20–39 age group. **Results:** The hospital discharge rate per 1,000,000 people was 3.08 in 2005 and 4.50 in 2021 for HCC, and 4.81 in 2005 and 1.92 in 2021 for cirrhosis. Comparing the early and later periods, values were higher for HCC (HDRR 1.13; 95% CI: 1.06–1.20) and lower for cirrhosis (HDRR 0.56; 95% CI: 0.51–0.60). The rate for the 20–39 age group was lower for the vaccinated compared with the unvaccinated cohorts overall (HDRR 0.53; 95% CI: 0.45–0.62), for HCC (HDRR 0.66; 95% CI: 0.53–0.82), and for cirrhosis (HDRR 0.41; 95% CI: 0.33–0.53). **Conclusions:** This study describes the important impact, after 25 years, of universal HBV vaccination in Spain: cirrhosis hospital discharge rate was reduced, and the vaccinated cohorts, compared with the unvaccinated cohorts in the 20–39 age group, had a lower hospital discharge rate of both HCC and cirrhosis.

## 1. Introduction

Hepatitis B virus (HBV) damages the liver through acute or chronic infection. The risk of progression from acute to chronic infection is inversely proportional to age on infection; thus, the risk is approximately 90% in newborns, 20% in children, and under 5% in immunocompetent adults [1]. Most of the disease burden is associated with chronic HBV infection, defined as HBV surface antigen (HBsAg) persistence in serum for at least 6 months.

The main consequences of chronic HBV infection are cirrhosis and hepatocellular carcinoma (HCC), which both lead to frequent hospitalization. In persons with chronic HBV infection referred to clinical centers, annual cirrhosis incidence is as high as 2–3% [2], while the annual risk of HCC is estimated to be under 1% and 2–3% for chronic HBV infection without and with cirrhosis, respectively [3]. The evolution of chronic HBV infection is determined by the interaction between virus replication and host immune response. Additional factors contributing to the progression to cirrhosis are mainly male sex [4,5], alcohol consumption, and coinfection with other hepatotropic viruses [6]. Meanwhile, the role of certain comorbidities (e.g., diabetes mellitus) in increasing the risk of HBV progression is controversial [7,8].

Worldwide, chronic HBV infection is an important cause of morbidity and mortality. The 2019 Global Burden of Disease (GBD) study [9] estimated that 316 million people were chronically infected and that HBV-related diseases resulted in 555,000 deaths (487,000–630,000). HBV was the leading cause of death from liver cancer and the third largest contributor to deaths from cirrhosis. HBV-related cirrhosis was responsible for 331,000 (279,000–392,000) deaths, and HBV-liver cancer was responsible for 192,000 (162,000–224,000) deaths [9]. The WHO estimates that 10.6 million people were living with hepatitis B in the European Region in 2022; of those living with hepatitis B, 16% had been diagnosed, and 12% of the diagnosed had received treatment [10].

In Spain, universal HBV vaccination for preadolescents and adolescents (aged 10–14 years) was introduced in 1991 in Catalonia and by 1996 in the remaining Spanish regions under the auspices of the first Recommended Immunization Schedule (RIS) of the Interterritorial Council of the National Health System [11,12]. By 2002, all Spanish regions had introduced universal vaccination for infants in the first year of life [13]; vaccination of 10–14-year-olds was maintained until 2014, by which time the RIS indicated immunization only of infants in the first year of life. Between 2005 and 2014, two cohorts were vaccinated: children under 1 year old and adolescents with pediatric hepatitis B vaccine; from 2014, vaccines were administered only in the first year of life. The vaccine used between 2014 and 2015 was the pediatric hepatitis B vaccine, but from 2015, the hexavalent vaccine containing antigen against hepatitis B was incorporated into the vaccination schedules. HBV vaccination coverage was high, at 75–90% in preadolescents and adolescents between 2003 and 2013 [14,15] and above 95% in infants in the first year of life [16]. The incidence of acute HBV decreased by 30.9% between 1997 (2.97/100,000 people)—the year following countrywide preadolescent and adolescent HBV vaccination—and 2018 (0.92/100,000 people) [17,18].

Given that vaccination is very efficacious in preventing HBV infection (80–100%) [6] and that the RIS has been shown to induce seroprotection levels greater than 95% in healthy infants, children, and young adults [19], we should expect the Spanish universal HBV vaccination program to have resulted in a reduced frequency of hospitalizations due to cirrhosis and HCC.

The aim of this study was to analyze, for Spain, the impact of universal HBV vaccination on chronic HBV-related hospital discharges over a 16-year period: from 2005 (9 years after countrywide preadolescent and adolescent HBV vaccination) to 2021 (19 years after universal vaccination of infants in the first year of life).

## 2. Materials and Methods

In this retrospective descriptive study from 2005 to 2021, we analyzed chronic HBV-related hospital discharges in the Spanish National Health System’s Minimum Basic Data Set (MBDS), an integrated medical record and data system containing patient hospitalization data.

To identify chronic HBV-related hospital discharges associated with cirrhosis and HCC, we searched through International Classification of Diseases (ICD) codes as follows: for primary diagnoses, 070, 155.0, 571.40, 571.41, 571.49, 571.5, 571.8, and 571.9 in ICD-9, and C22.0, K72.11, K72.91, K73.9, K74.0, K74.2, K74.6, K74.69, and K75.3 in ICD-10; and for secondary diagnoses, 070.32 and 070.33 in ICD-9, and B18.0 and B18.2 in ICD-10 (Supplementary Table S1).

Excluded were hospital discharges associated with secondary hepatitis C virus, i.e., ICD-9 codes 070.41, 070.44, 070.51, 070.54, 070.70, 070.71, and V02.62, and ICD-10 codes B17.1, B17.10, B17.11, B18.2, B19.2, B19.20, and B19.21.

The following sociodemographic data were collected: sex, age, region of residence, admission date, discharge date, and type of discharge. Population data were obtained from the National Institute of Statistics, and medical data from the MBDS.

In order to assess the evolution in chronically infected HBV patients, ICD-9 and ICD-10 codes for HBV-related hospital discharge rates as a measure of the burden of disease were used. HBV-related hospital discharge rates per 100,000 person-years or 1,000,000 person-years were calculated overall and by principal diagnosis (HCC and cirrhosis), sex, specific age groups, and birth cohort. The early (2005–2013) versus later (2014–2021) vaccination periods were compared by calculating the hospital discharge rate ratio (HDRR) and 95% confidence interval (CI) values.

HDRRs and 95% CI values were also calculated for the 20–39 age group since this age group encompassed persons aged 12 (the average age at which vaccination began) in 1991–1996 and so could be used as a proxy of vaccinated cohort in 2005–2021 for comparison with an unvaccinated cohort (Supplementary Table S2). We defined the vaccinated cohorts and the unvaccinated cohorts, taking into account the year of immunization program implementation, the date of birth, and the region of residence of the patients. Vaccination cohorts were defined as people born after the years 1979–1985, depending on the region of residence.

Incidence trends were calculated using the chi-square test for trend.

Analyses were performed using the SPSS v.25 statistical package (IBM Inc., Armonk, NY, USA), R v.4.3.2 statistical software (Vienna, Austria), and OpenEpi v.3.01 (Atlanta, GA, USA).

## 3. Results

In the MBDS over the 16-year study period (2005–2021), 6919 chronic HBV-related discharges were recorded: 83.2% (5754) men and 16.8% (1165) women. The male-to-female ratios were 8.9 for HCC and 2.9 for cirrhosis. HCC and cirrhosis accounted for 3908 and 2835 hospital discharges, and excluded from the analysis were 176 cases with unspecified chronicity.

**Figure 1:**
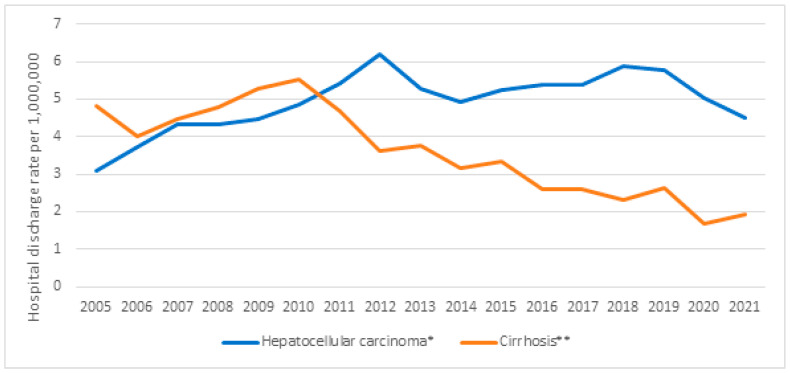
Total hospital discharge rate values for cirrhosis and hepatocellular carcinoma as the main diagnoses in chronic hepatitis B infections (2005–2021). * Significant increasing trend, *p* = 0.006. ** Significant decreasing trend, *p* < 0.001.

**Figure 2:**
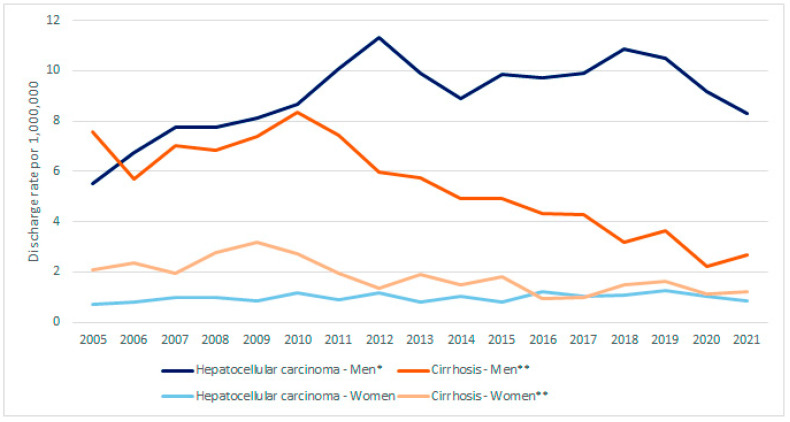
Hospital discharge rate values by sex for cirrhosis and hepatocellular carcinoma as the main diagnoses in chronic hepatitis B infection (2005–2021). * Significant increasing trend, *p* = 0.006. ** Significant decreasing trend, *p* < 0.001.

Figure 1 and Figure 2 show the hospital discharge rates per 1,000,000 people for HCC and cirrhosis, overall and by sex, respectively. The values for HCC, at 3.08 in 2005 and 4.50 in 2021, showed a significant increasing trend (*p* = 0.006), and the values for cirrhosis, at 4.81 in 2005 and 1.92 in 2021, showed a significant decreasing trend (*p* < 0.001). Observed in men were a significant upward trend for HCC (*p* = 0.006), with values of 5.51 in 2005 and 8.31 in 2021, and a significant downward trend for cirrhosis (*p* < 0.001), with values of 7.58 in 2005 and 2.67 in 2021. As for women, a significant downward trend was only observed for cirrhosis (*p* < 0.001), with values of 2.11 in 2005 and 1.20 in 2021.

Table 1 compares HCC and cirrhosis hospital discharge rates for the early (2005–2013) versus later (2014–2021) periods. HCC values were higher in the later period, both overall (HDRR 1.13; 95% CI: 1.06–1.20) and in men (HDRR 1.14; 95% CI: 1.07–1.22); the HCC values for women were also higher, but not to a statistically significant degree (HDRR 1.12; 95% CI: 0.92–1.36). Values for cirrhosis were lower during the 2014–2021 period, overall and in both men and women (HDRR 0.56; 95% CI: 0.51–0.60; HDRR 0.55; 95% CI: 0.50–0.60 and HDRR 0.59; 95% CI: 0.50–0.68, respectively).

**Figure 3:**
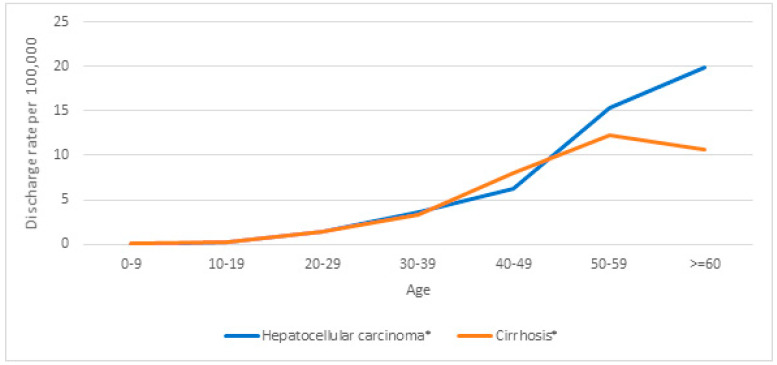
Hospital discharge rate ratio (HDRR) values by age group for cirrhosis and hepatocellular carcinoma as the main diagnoses in chronic hepatitis B infection (2005–2021). * Significant increasing trend, *p* < 0.001.

**Figure 4:**
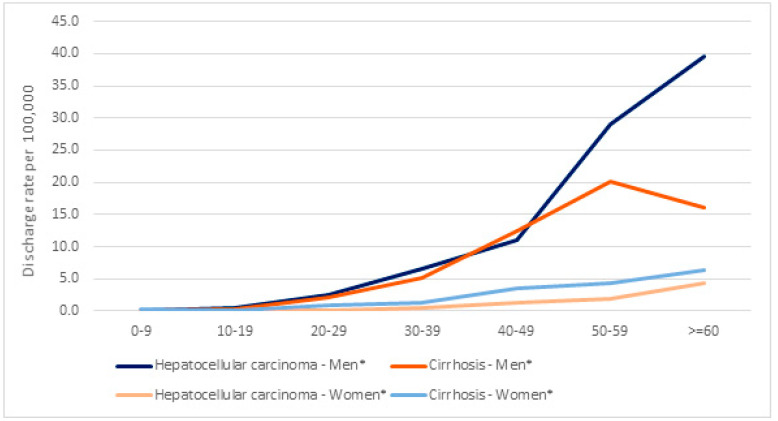
Hospital discharge rate ratio (HDRR) values by sex for cirrhosis and hepatocellular carcinoma as the main diagnoses in chronic hepatitis B infection (2005–2021). * Significant increasing trend, *p* < 0.001.

**Figure 5:**
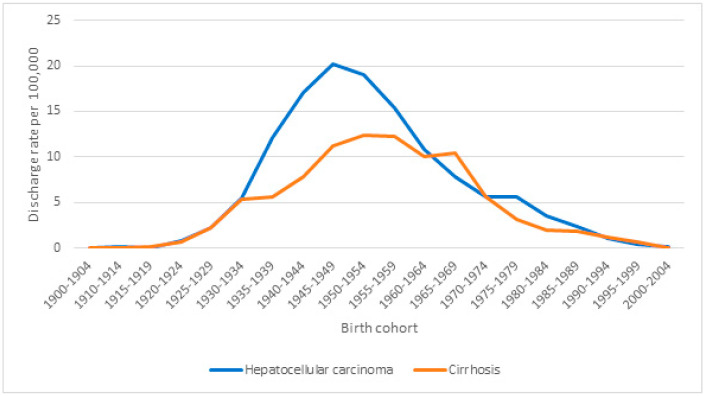
Hospital discharge rate ratio (HDRR) values by birth cohort for cirrhosis and hepatocellular carcinoma as the main diagnoses in chronic hepatitis B infection (2005–2021).

Figure 3 and Figure 4 show hospital discharge rates per 100,000 people for HCC and cirrhosis according to age group and sex, respectively, showing significant increasing trends (*p* < 0.001) overall, by age, and for men and women separately. Figure 5 shows hospital discharge rates per 100,000 people for HCC and cirrhosis by birth cohort. The highest values observed were 202.2 for HCC in the 1945–1949 age cohort and 12.4 for cirrhosis in the 1950–1955 cohort.

Finally, Table 2 shows hospital discharge rates for HCC and cirrhosis for the 20–39 age group according to cohorts (vaccinated and unvaccinated). Values were lower for the vaccinated cohorts overall (HDRR 0.53; 95% CI: 0.45–0.62), for HCC (HDRR 0.66; 95% CI: 0.53–0.82), and for cirrhosis (HDRR 0.41; 95% CI: 0.33–0.53).

## 4. Discussion

The highest hospital discharge rate for chronic HBV-related discharges occurred in men: for HCC in men older than 60 years and for cirrhosis in men aged 50–59 years. The male-to-female ratio for HCC was 8.9, and for cirrhosis was 2.9.

Sex differences in HCC and cirrhosis incidence have been reported by different authors, underpinning the importance of separate analyses for men and women. Ruggieri et al. [20] pointed to sex/gender disparity mechanisms in hepatitis viral infection progression and outcomes, contributed by, in addition to viral factors, the synergistic action of male and female sex hormones and immune responses.

HBV-related HCC affects men more frequently than women, with reported male-to-female ratios of 2 to 7 [8,20,21,22,23,24]. The HCC ratio was 8.9 in our study, suggesting not only that HCC incidence is higher in men but also that men are more frequently hospitalized with HCC. In an Italian study by Stroffolini et al., the male-to-female ratio was 6.8 [24]. HBV-related cirrhosis is also more common in men than in women, as shown by the 2.9 reported by us. Findings corroborating this male-to-female ratio have been reported elsewhere: 3.6 was reported by an Italian hospital study of patients admitted with hepatitis B-related liver cirrhosis [24], and 3.3 was reported by a Chinese hospital study of patients aged 18 years and older [23].

In the period 2005–2021, the overall HDRR for HBV-related cirrhosis fell by 60.1%, and this decrease was greater in men than in women. While the hospital discharge rate increased from 2005 to 2009, 2010 marked a watershed, with a notable decrease since then. In contrast, the overall HDRR for HBV-related HCC increased by 46.1% in the same period, by 50.8% in men and 16.9% in women. The increase was notable from 2005 to 2010, but the trend stabilized from 2011 to 2021. For the entire Spanish population, we found that the cirrhosis hospital discharge rate showed a decreasing trend in both men and women, while the HCC hospital discharge rate showed an increasing trend for men but not for women. We are dealing with discharges of patients with HBV infection, and it seems that the evolution of this infection can be an explanation for the change observed, but we cannot exclude that other demographic and behavioral factors or other metabolic diseases might have had a role [1,7,8,25]. It is also possible that the decrease in 2020 and 2021 could be related to low hospitalization rates of non-COVID-19 diseases observed in Spain and other countries [26,27].

The effect of antiviral therapies on HBV-related HCC incidence has been investigated by other authors. Papatheodoridis et al. [28], in a study carried out in different European countries, found that the HCC risk in patients older than 50 years persisted 5 years after antiviral treatment and also that older age was independently associated with HCC development. In a modeling study, Negro et al. [29] observed that while cirrhosis incidence showed a decreasing trend, HCC incidence increased. These authors suggested that current therapies are not curative and that some treated individuals still develop HCC.

Regarding the effect of vaccination in the 20–39 age group (which includes persons aged 12 in 1991–1996), we found that the HDRR was lower in the vaccinated cohort overall (HDRR 0.53; 95% CI: 0.45–0.62), for HCC (HDRR 0.66; 95% CI: 0.53–0.82), and for cirrhosis (HDRR 0.41; 95% CI: 0.33–0.53). In the 40–49 age group and in people older than 50 years, the HCC value increased, and although more important in men, the increase was also observed in women.

In the Spanish study by Hernando et al. [17] covering the period 1997–2007, cases with chronic HBV as the main diagnosis fell overall by 3.3%, but more so in men and in the 35–44 age group.

In the case of HBV-related cirrhosis, Huang et al. [30] reported that the overall burden in children and adolescents fell significantly over the period 1990–2019, with slightly lower rates in males than in females. In a study carried out by Sagnelli et al. [31], 23 years after the introduction (2001 to 2014) of universal HBV vaccination in Italy, while cirrhosis incidence decreased from 17.5% to 5.2% in the under 33 age group, it increased from 39.2% to 49.3% in the 60+ age group, while HCC increased in the 34–59 age group.

Chang et al. [32], in a Taiwanese study investigating whole HCC incidence for the period 1983–2011, reported a rate ratio for the 20–26 age group of 0.42 (95% CI: 0.32–0.56), lower than the 0.66 found in our study for the 20–39 age group; a plausible explanation might be that HCC incidence in Taiwan before starting the HBV vaccination program (in 1992) was much higher than in Spain and, therefore, the impact of infant vaccination is greater [33]. Interestingly, in that same study, as occurred in our study, the hazard rate for whole HCC in the vaccinated cohorts was lower in the latter compared with the earlier cohorts.

In another Taiwanese study, Chiang et al. [34] compared the evolution of whole HCC incidence over the period 1979–2018 according to birth cohort, comparing the periods before and after vaccination introduction and age groups. They found that HCC mortality and incidence both fell dramatically after a national chronic hepatitis antiviral therapy program was launched in 2004, that chronic liver disease and HCC mortalities started to fall 5 years before the program introduction, and that HCC incidence decreased in both sexes but more so in men than women. The authors concluded that both HBV vaccination and antiviral therapy programs were associated with a substantially reduced chronic liver disease burden since 1979 and suggested that a dual strategy of universal vaccination and antiviral therapy would be useful in reaching the World Health Organization (WHO) goal of eliminating HBV by 2030.

The incidence rate of HCC increases with advanced age worldwide [32], as has also been observed in the present study, but interestingly, the hospital discharge rates for HCC were lower in vaccinated cohorts than in unvaccinated cohorts.

The impact of HBV on HCC incidence is useful to assess the importance of vaccination. Studies have been carried out in Taiwan, China, and Alaska, showing that the vaccine prevented [32,35] or even eliminated [36] the development of HCC. However, more studies are needed to assess in different settings the contribution of HBV vaccination to the reduction of HCC, both in developed and developing countries.

Li et al. [37], in a study of HCC trends according to the 2007 GBD study, reported that HBV-related liver cancer incidence fell significantly in children, adolescents, and young adults in countries with national HBV vaccination programs but increased in the general population in countries without HBV vaccination programs. In their analysis of the 2019 GBD study, Cortesi et al. [38] reported that although 83.3% (20 of 24) of European Union and European Economic Area countries had implemented universal childhood HBV vaccination programs that achieved 90% coverage, the HBV-related HCC burden did not show any significant improvement between 2010 and 2019; this was because HCC was more common in older adults, with a median age at diagnosis of around 60 years, whereas the first neonatal vaccination programs began in around 1990.

McMahon et al. [36], in an analysis of the impact of a universal HBV immunization program for native newborn Alaskans, found, after 25 years, that whole HCC incidence in persons aged under 20 years had fallen from 3 to 0 per 100,000 from 1984–1988 to 1995–1999.

The WHO [39] goal for viral hepatitis elimination by 2030 as a public health problem establishes targets of 90% and 65% reductions in chronic HBV infection incidence and mortality, respectively. Since deaths in China due to HBV-related liver disease account for around 30% of worldwide HBV mortality, China will be a key contributor to the WHO’s goal of eliminating HBV as a public health threat by 2030. In the study by Cao et al. [40] carried out in China from 1990 to 2019, HBV-related deaths for acute hepatitis, cirrhosis, and other chronic liver diseases (including liver cancer) decreased by 74.8%, 34.7%, and 23.3%, respectively; however, from 1990 to 2019, HBV-related deaths increased in the 70+ age group, while from 2015 to 2019, deaths from HBV-related liver cancer increased by 7.05%, mainly in the 50–69 age group.

Despite the WHO goal for viral hepatitis elimination, the annual global deaths from HBV are projected to increase by 39% from 2015 to 2030 if the status quo remains [41]. In our study, it was observed that the overall HDRR for HBV-related HCC increased from 2005 to 2010 and remained stable from 2010 to 2021.

Reinforcing messages about the importance of vaccination and screening for infection [42,43] in the general population and risk groups is crucial because there are some infected people who do not know they are infected. In a community seroepidemiological study carried out in Spain, 0.5% of participants had a VHB chronic infection, and less than half were aware of their condition [44]. In another study carried out also in Spain among eligible people living with HIV, only 9% completed the HBV vaccination. There is an urgent need to improve HBV vaccination to reach the WHO call for hepatitis B elimination [45]. Some authors recommend that HBV serological testing should be performed at least once in all adults, regardless of any HB risk exposure [46].

As it has been recently stated, one of the main areas of action to advance in the implementation of a public health approach to viral hepatitis and achieve 2030 targets is the improvement of country data and accountability for viral hepatitis [10]. In this way, it is important to assess and quantify, using available hospitalization data, whether the burden of severe disease due to hepatitis B has decreased [31,37].

Globally, major progress on immunization programs has been achieved with three doses of immunization coverage during infancy, and countries that have adopted this schedule have observed a reduction in carrier state as well as complications from HBV, including HCC [19]. The most straightforward approach involves administering the vaccine to all newborns. The available evidence suggests that the goal of achieving global HBV elimination by 2030 faces some challenges, especially in some low-income countries. In the African region, vaccine coverage is very low, and in low and middle-income countries, access to viral hepatitis testing and antiviral treatment needs to be improved significantly [47]. Despite advancements in preventive measures and prevalence goals for HBV in specific regions, research shows that diagnostic rates and accessibility to treatment should be improved.

Key barriers to achievement of hepatitis B elimination are derived from the fact that HBV infection can become chronic, impacting the lifespan of the patient and requiring monitoring throughout the course of the disease. Moreover, there is currently no cure, and therefore, there remains a residual risk of liver cancer [48]. To achieve hepatitis B, curative treatment support for further research and drug development is needed [49].

The prevention and treatment of hepatitis B require worldwide cooperation and effort. WHO in 2017 proposed five major interventions to reduce the threat of viral hepatitis, including the prevention of mother-to-child transmission; vaccination; injection, blood, and surgical safety; harm reduction services for people who inject drugs; and active antiviral treatment [50].

Beyond the impact on health, the economic benefits of universal vaccination have been documented by studies of countries at different levels of development [51,52,53].

In a study carried out in Italy [52], it was demonstrated that the return on investment and the benefit-to-cost ratios are >1 for the first thirty years of immunization onset with a net saving of EUR 396,494,926 from the National Health Service perspective and EUR 482,577,670 from the societal perspective. In Israel, early cost-effectiveness modeling of universal hepatitis B vaccination estimated a USD 690,000 health services benefit related to HCC cost over 45 years [54].

Investment in hepatitis B elimination is estimated to provide cost savings in the Philippines by 2024 and in Vietnam by 2027. By 2035, for every dollar spent on hepatitis B elimination activities, there would be an estimated return of USD 2.23 in the Philippines and USD 1.70 in Vietnam [55,56].

Cost-effective strategies aimed at increasing birth dose vaccination coverage in low-resource settings include controlled temperature chain storage strategies, as well as the utilization of prefilled delivery devices [57].

Although there is a high degree of heterogeneity among regions and countries, global hepatitis B morbidity and mortality rates decreased significantly from 1990 to 2019 targets [26,27]. Many countries are being held back due to a lack of funding, yet it is time to finance the fight against hepatitis HBV by upgrading diagnosis and treatment rates [58]. Therefore, without adequate and equitable approaches to further reduce morbidity and mortality, achieving the WHO goal of the elimination of viral hepatitis by 2030 seems very difficult to achieve or even unachievable, as stated by some authors [41,58,59,60].

Greater commitment is needed from all stakeholders involved in hepatitis B elimination, from governments to international institutions, donors, and the population in general. However, for countries currently not funding hepatitis B elimination activities, the affordability of investment at the expense of competing priorities needs to be addressed. The investment framework presented by Howell et al. [55] identifies key activities to achieve hepatitis B elimination targets and solutions to funding shortfalls to achieve maximal impact. Financial support from international agencies and donors for elimination activities is vital for many lower and middle-income countries to successfully achieve elimination targets.

Focused efforts on investment and implementation for equitable treatment in key high-burden regions are essential to meet the 2030 goals for viral elimination [10,61]. Raising awareness and education on hepatitis elimination among health-care providers, as well as policy makers and other stakeholders, is a priority to accelerate progress, mainly in high-burden regions [59].

After knowing the burden of cirrhosis and HCC-related hospitalizations in Spain, a second step should be to carry out an economic study with these data.

This study has a number of limitations. First, since we used retrospective population-based data, vaccination status could only be a proxy based on immunization schedule implementation, birth cohort, and region of residence on discharge, as other authors have done [32]. In addition, study subjects may have been hospitalized for both cirrhosis and HCC during the study period. Second, it was not possible to separately estimate the contribution of vaccination and the impact of antiviral treatment, as, although HBV treatment started in Spain around 2006 [62], this information was not incorporated in this study. It has been stated that optimal control strategies for preventing hepatitis B infection and reducing chronic liver disease incidence include vaccination and treatment [34,40,63]. Third, although global migration is also an important determinant of the burden of chronic hepatitis B [33], we have not taken into account this information because it was not available until 2016; however, the percentage of the immigrant population in Spain during the study period was quite similar: 9% in 2005, and 11.5% in 2021.

The main strength of our study is the universal data coverage due to the fact that all discharges of the National Health System (which accounts for 96% of the population [64]) are recorded, meaning that there is a very low risk of underestimating the hepatitis B hospitalization burden.

## 5. Conclusions

It is important to assess and quantify whether the burden of severe disease due to hepatitis B has decreased as a result of the implementation of immunization programs. Our study confirms an important impact after 25 years of universal HBV vaccination in Spain. Although the protective effect was higher for cirrhosis than for HCC, vaccinated cohorts showed a lower hospital discharge rate of both hepatitis B-related cirrhosis hospitalization and hepatitis B-related HCC hospitalization than unvaccinated cohorts.

## Figures and Tables

**Table 1:** Comparative hospital discharge rate ratio (HDRR) values due to HBV-related cirrhosis and hepatocellular carcinoma for the early and later periods following the introduction of the hepatitis B vaccine.

	Hospital Discharges	Hospital Discharges per 1,000,000	HDRR (CI 95%)	*p*-Value
**All hospitalizations**				
**Overall**				
2005–2013	3821	9.19	Ref.	
2014–2021	2922	7.79	0.85 (0.81–0.89)	<0.001
**Male**				
2005–2013	3151	15.36	Ref.	
2014–2021	2470	13.43	0.87 (0.83–0.92)	<0.001
**Female**				
2005–2013	670	3.18	Ref.	
2014–2021	452	2.36	0.74 (0.66–0.84)	<0.001
**Hepatocellular carcinoma**				
**Overall**				
2005–2013	1933	4.65	Ref.	
2014–2021	1975	5.26	1.13 (1.06–1.20)	<0.001
**Male**				
2005–2013	1737	8.45	Ref.	
2014–2021	1776	9.65	1.14 (1.07–1.22)	<0.001
**Female**				
2005–2013	196	0.93	Ref.	
2014–2021	199	1.04	1.12 (0.92–1.36)	0.27
**Cirrhosis**				
**Overall**				
2005–2013	1888	4.54	Ref.	
2014–2021	947	2.52	0.56 (0.51–0.60)	<0.001
**Male**				
2005–2013	1414	6.89	Ref.	
2014–2021	694	3.77	0.55 (0.50–0.60)	<0.001
**Female**				
2005–2013	474	2.25	Ref.	
2014–2021	253	1.32	0.59 (0.50–0.68)	<0.001

Ref.: reference category.

**Table 2:** Comparative hospital discharge rate ratio (HDRR) values due to cirrhosis and hepatocellular carcinoma for the hepatitis B vaccinated and unvaccinated 20–39 age cohorts.

	Hospital Discharges	Hospital Discharges per 1,000,000	HDRR (CI 95%)	*p*-Value
**Overall**				
Vaccinated cohorts	233	1.05	0.53 (0.45–0.62)	<0.001
Unvaccinated cohorts	438	1.98	Ref.	
**Hepatocellular carcinoma**				
Vaccinated cohorts	138	0.62	0.66 (0.53–0.82)	<0.001
Unvaccinated cohorts	209	0.94	Ref.	
**Cirrhosis**				
Vaccinated cohorts	95	0.43	0.41 (0.33–0.53)	<0.001
Unvaccinated cohorts	229	1.03	Ref.	

Ref.: reference category.

## Data Availability

This study involved the use of patient data owned by third-party organizations. The MBDS is a set of clinical-administrative data of hospitalizations that collects sociodemographic, clinical, and administrative patient data from the National Health System discharge reports. It is hosted by the Ministry of Health (https://www.sanidad.gob.es/en/estadEstudios/estadisticas/cmbdhome.htm (accessed on 22 January 2024)). Data are available upon request to the Ministry of Health, including a confidentiality agreement, with the commitment not to share data with third parties. To gain access to the data, researchers need to fill in an official form explaining the research aims and expected results.

## Supplementary Materials

The following supporting information can be downloaded at: https://www.mdpi.com/article/10.3390/vaccines12111254/s1, Table S1: Chronic hepatitis B-related diagnostic codes; Table S2: Ages in 2005–2021 of persons who were 12 years old in 1991–1996.
