# Supplementary material for: Hepatitis B Virus-Related Cirrhosis and Hepatocellular Carcinoma Hospital Discharge Rates from 2005 to 2021 in Spain: Impact of Universal Vaccination
Source: Vaccines (Basel). 2024 Nov 4;12(11):1254. doi: 10.3390/vaccines12111254 (PMC11598889; doi:10.3390/vaccines12111254)
Supplement: Supplementary file 1 [file vaccines-12-01254-s001.zip › Suplementary tables.pdf]

Supplementary Table S1. **Chronic hepatitis B-related diagnostic codes.**

Main diagnoses

ICD-9:

070 - Viral hepatitis

155.0 - Malignant neoplasm of liver

571- Chronic liver disease and cirrhosis

- 571.40 – Chronic hepatitis, unspecified
- 571.41 – Chronic persistent hepatitis
- 571.49 – Other chronic hepatitis
- 571.5 – Cirrhosis of liver without mention of alcohol
- 571.8 – Other chronic nonalcoholic liver disease
- 571.9 – Unspecified chronic liver disease without mention of alcohol

ICD-10:

C22.0 – Liver cell carcinoma

K72 - Hepatic failure, not elsewhere classified

- K72.1 – Chronic hepatic failure
- K72.9 – Hepatic failure, unspecified

K73 – Chronic hepatitis, not elsewhere classified

K74.0 – Fibrosis and cirrhosis of liver

- K74.2 – Hepatic fibrosis with hepatic sclerosis
- K74.6 – Other and unspecified cirrhosis of liver

K75 - Other inflammatory liver diseases

- K75.3 – Granulomatous hepatitis, not elsewhere classified

Secondary diagnoses

ICD-9:

070 - Viral hepatitis

- 070.32 – Chronic viral hepatitis B without delta-agent
- 070.33 – Chronic viral hepatitis B with delta-agent

ICD-10:

B18 - Chronic viral hepatitis

- B18.0 – Chronic viral hepatitis B with delta-agent
- B18.1 – Chronic viral hepatitis B without delta-agent

Supplementary Table S2. **Ages in 2005-2021 of persons who were 12 years old in 1991-1996.**

		Year of the study																
		**2005**	**2006**	**2007**	**2008**	**2009**	**2010**	**2011**	**2012**	**2013**	**2014**	**2015**	**2016**	**2017**	**2018**	**2019**	**2020**	**2021**
Year of immunization program implementation	**1991**	26	27	28	29	30	31	32	33	34	35	36	37	38	39	40	41	42
	**1992**	25	26	27	28	29	30	31	32	33	34	35	36	37	38	39	40	41
	**1993**	24	25	26	27	28	29	30	31	32	33	34	35	36	37	38	39	40
	**1994**	23	24	25	26	27	28	29	30	31	32	33	34	35	36	37	38	39
	**1995**	22	23	24	25	26	27	28	29	30	31	32	33	34	35	36	37	38
	**1996**	21	22	23	24	25	26	27	28	29	30	31	32	33	34	35	36	37

Note: The average age at which vaccination began was 12 years.
